# One for all

**DOI:** 10.7554/eLife.14150

**Published:** 2016-02-11

**Authors:** Duncan T Odom

**Affiliations:** Cancer Research UK Cambridge Institute, University of Cambridge, Cambridge, United KingdomDuncan.Odom@cruk.cam.ac.uk

**Keywords:** transcription, transcription factor binding, evolution, genome evolution, comparative genomics, cis-regulatory sequences, Chicken, <i>D. melanogaster</i>, Human, Mouse, Rat

## Abstract

Using a common analysis pipeline to compare data from three major lineages of complex eukaryotes reveals that transcription seems to evolve at a common rate.

**Related research article** Carvunis A-R, Wang T, Skola D, Yu A, Chen J, Kreisberg JF, Ideker T. 2015. Evidence for a common evolutionary rate in metazoan transcriptional networks. *eLife ***4**:e11615. doi: 10.7554/eLife.11615

**Image** Genome sequences evolve more rapidly in mammals (red) than in birds (yellow) and insects (blue): x-axis is evolutionary separation from reference species; y-axis is a measure of genomic similarity

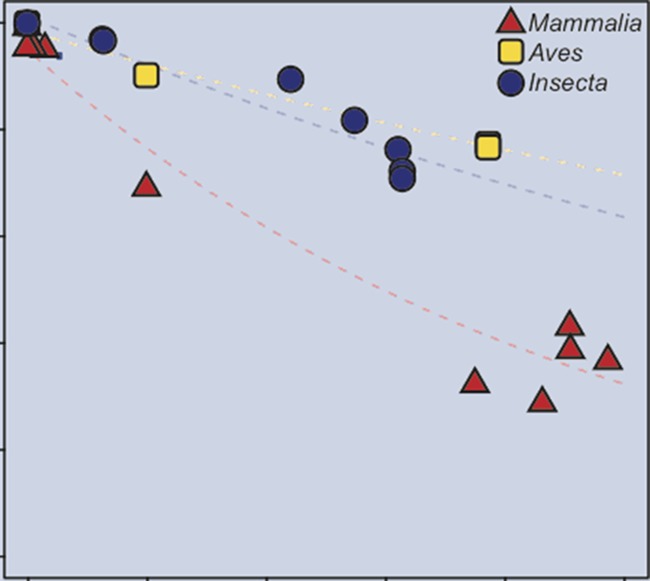

There is an old saying in computational circles that researchers in bioinformatics would rather use someone else’s toothbrush than use someone else’s code. One example of this adage being true can be seen in previous attempts to compare the rates at which differences in the mechanisms that control DNA accumulate in different species and lineages.

The information contained in DNA is first accessed by dedicated proteins called transcription factors (TF) that bind to preferred sequence of bases in the DNA. This sequence is typically short, between 8 and 20 bases in length (Vaquerizas et al., 2009), although some can be as long as 35 bases (Filippova et al., 1996). After transcription factor binding has taken place, the basal transcription machinery and its associated complexes open the region’s chromatin and begin transcribing DNA into RNA. These crude transcripts must undergo extensive processing and maturation before they can be exported to the cytoplasm as mature messenger RNA (mRNA). Understanding the rate at which all these steps (notably transcription factor binding and the production of mRNA) change during evolution is a long-standing goal in genetics (Wray, 2007; Wittkopp and Kalay, 2012).

Technically, it is (relatively) easy to map all the contacts between the transcription factors and the DNA, and also to map all the mRNA molecules, in a biological sample using high-throughput sequencing technologies. A number of research groups have compared the amount of transcription factor binding in many species of flies and mammals (He et al., 2011; Paris et al., 2013; Schmidt et al., 2010; Ballester et al., 2014). Based on this work it seemed as if transcription factor binding evolved rapidly in mammalian tissues (Weirauch and Hughes, 2010), but only very slowly in fruit flies (He et al., 2011). However, it can be difficult to compare the first results generated in an entirely novel field of study because different groups often use very different approaches. And in this case this difficulty is further compounded by the toothbrush issue.

Now, in eLife, Trey Ideker and colleagues at the University of California San Diego – including Anne-Ruxandra Carvunis, Tina Wang and Dylan Skola as joint first authors – report that they used a new analysis pipeline to study the raw data for more than 25 species of complex eukaryotes across three animal lineages (mammals, birds and insects) that previously had only been studied in isolation (Carvunis et al., 2015). In other words, they have cleaned everyone’s teeth with the same toothbrush. Moreover, their pipeline could be tweaked to vary the analysis parameters for all the datasets across three lineages at once, thus allowing them to make like-with-like comparisons.

This intellectual scrubbing resulted in two major insights. First, it appears that transcription factor binding (which dictates the function of the genome) and mRNA both evolve at a shared (and perhaps even fundamental) rate in complex eukaryotes. This result is somewhat surprising since most evolutionary geneticists think that the mechanisms that influence genome or functional evolution for the lineages studied by Carvunis et al. are radically different.

Second, particularly in mammals, the evolution of the genome sequence en masse is much more rapid than the evolution of transcription factor binding and transcription. This disconnect may be linked to the instability of the large number largely-silent repeat elements in mammalian genomes, and/or to the fact that insects and birds have more stable genomes.

Moreover, Carvunis et al. have powerfully demonstrated why it is important for all of us in the functional genomics community to meticulously curate our raw data and to make it readily available for others to analyse. None of the insights reported in this work would have been possible without easy access to carefully annotated sequencing reads from the original studies.
